# Association of CYPA1 gene polymorphism with plasma nitric oxide levels in COPD

**DOI:** 10.1186/1471-2164-15-S2-P9

**Published:** 2014-04-02

**Authors:** Ashrafunnisa Begum, Venkateshwari, Fazal Mohammed, A Jyothy

**Affiliations:** 1 Institute of Genetics and Hospital for Genetic Diseases, Osmania University, Hyderabad 500016, India; 2 Vita Food Company, Dammam, KSA

## Background

Chronic obstructive pulmonary disease (COPD) is characterized by irreversible airflow limitation, abnormal permanent distal air-space enlargement and emphysema in the lungs. Increased oxidative burden in COPD is because of both directly a result of smoking and indirectly by the release of increasing amount of ROS from airways leukocytes. The CYP1A1 gene modifies the phase I enzyme aryl hydrocarbon hydroxylase (AHH) belonging to the cytochrome P450 system that plays a major role in the metabolism of exogenous toxins generated by cigarette smoke [1]. The aim of the present study is to assess the role of CYP1A1 gene polymorphism and to measure the plasma nitric oxide levels in the etiology of COPD in South Indian Population from Andhra Pradesh.

## Materials and methods

A cohort of 250 clinically and spirometrically confirmed COPD patients referred to Government Chest Hospital, Hyderabad and an equal member of age and sex matched control subjects were included in the present study. Genotyping of CYP1A1 gene polymorphism was done by PCR – RFLP method followed by agarose gel electrophoresis. The plasma nitrite levels were estimated by spectrophotometric method. Appropriate statistical methods were applied to test for the significance of the results.

## Results

**Figure 1:**
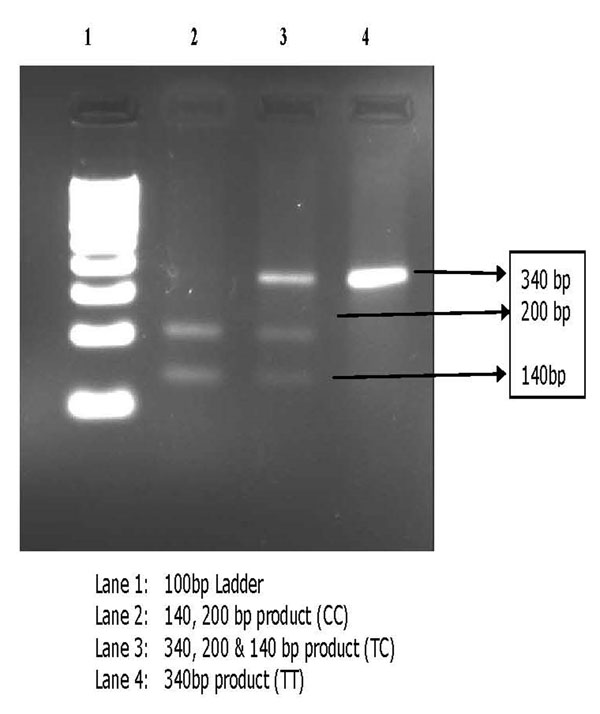
Agarose Gel Electrophoresis showing the Amplification of 340 bp, 200 bp and 140 bp product After Restriction enzyme digestion by (MspI).

Genotypic distribution of CYP1A1 gene polymorphism revealed 36.8% of TT, 50.8% of TC and 12.4% of CC genotypes in COPD patients and 44.8% of TT, 48.4% of TC and 6.8% of CC in control subjects (Figure 1). There is an increased frequency of CC genotype and C allele in the COPD patients compared to control subjects (*X*^2^ = 4.51; p=0.03; OR=1.94; 95% CI = (1.044-3.605) and (*X*^2^=5.12; p=0.02; OR=1.35; 95% CI=(1.041-1.758). (Table 1). The study carried out from North Indian population have shown that CYP1A1,-3801 polymorphism was significantly associated with COPD [2]. The mean levels of plasma nitrite levels were also found to significantly elevated in COPD patients (2.715 ± 1.552) in comparison with their respective controls (1.123 ± 0.699) (p<0.001) (t value = 2.6449).

**Table 1:** Comparison of genotypes and alleles of CYPA1-3801 T /C in COPD and Controls

X^2^	p Value	Odds Ratio 95% CI	Genotype/Allele
TT Vs TC + CC	3.31	0.06	0.71(0.5015-1.026)
TC Vs TT+CC	0.28	0.59	1.10(0.7752-1.563)
CCVs TC + TT	4.51	0.03	1.94(1.044-3.605)
C Vs T	5.12	0.02	1.35(1.041-1.758)
T Vs C	5.12	0.02	0.73(0.56-0.960)

## Conclusions

The present study indicates the association of CYP1A1 gene polymorphism and plasma nitric oxide levels in the etiology of COPD patients of South Indian population from Andhra Pradesh.
